# Hepatic sarcoidosis: a case series

**DOI:** 10.11604/pamj.2016.24.209.7980

**Published:** 2016-07-08

**Authors:** Rym Ennaifer, Shema Ayadi, Hayfa Romdhane, Myriam Cheikh, Houda Ben Nejma, Wassila Bougassas, Najet Bel Hadj

**Affiliations:** 1 Department of Hepato-gastro-enterology, Mongi Slim Hospital, Tunis, Tunisia

**Keywords:** Sarcoidosis, liver, granuloma, cholestasis, corticosteroids

## Abstract

Sarcoidosis is a systemic non caseous granulomas disease. Liver is a common location but usually asymptomatic. Evidence based guidelines for this location treatment is lacking and the effect of corticosteroids may be inadequate. The aim of our study was to describe the clinical, biochemical, radiological and therapeutic features of seven patients with systemic sarcoidosis and liver involvement. A retrospective and descriptive monocentric study, over 3 years, including seven patients with systemic sarcoidosis and liver involvement. We included 5 women and 2 men with an average age of 43 years. Hepatic localization revealed sarcoidosis in 5 cases. Hepatomegaly was observed in all patients as well as abnormal serum liver function test reflected by anicteric cholestasis. Liver biopsy, showed in all granulomatous lesions consistent with sarcoidosis and severe fibrosis in 2 cases. Extra-hepatic manifestations were present in all patients represented mainly by pulmonary location. All patients were treated, five by corticosteroid and two with ursodeoxycholic acid (UDCA). Complete response was observed in one case, partial response in another case and corticosteroid refractoriness in one case. In two cases, corticosteroid therapy was introduced for less than 1 month, not allowing assessment of response. Antimalarials in combination with UDCA were used successfully in a patient with steroid-resistant liver disease. Liver involvement can reveal systemic sarcoidois. Given the risk of progression to severe liver disease, it must be screened in all patients with systemic sarcoidosis. Treatment is not systematic, and still based on corticosteroid therapy. In the absence of prospective randomized controlled trials, the efficacy of UDCA need to be proven.

## Introduction

Sarcoidosis, a systemic disease of unknown etiology, is characterized by the presence of non caseous granulomas in the affected organs. Although it is revealed usually by mediastinal and pulmonary localization, the disease may affect any organ. Liver is a common location, approximately 50-80% of patients with systemic sarcoidosis have hepatic involvement at liver biopsy [1]. It is usually asymptomatic and is manifested by nonspecific biochemical disturbances; therefore it remains undiagnosed in many cases. Liver infiltration by granulomas can be responsible for anicteric cholestasis, however, only a minority of patient progress to portal hypertension, cirrhosis and liver failure. Exceptionally, liver involvement may results in Budd-Chiari syndrome (BCS). Evidence based guidelines for treatment of liver sarcoidosis is lacking and the effect of corticosteroids may be inadequate [2]. The aim of our study was to describe the clinical, biochemical, radiological and therapeutic features of seven patients with systemic sarcoidosis and liver involvement.

## Methods

A retrospective and descriptive study, including seven patients with systemic sarcoidosis and liver involvement, diagnosed between January 2011 and December 2014 in the department of Hepatology, Mongi Slim University Hospital, Tunisia. The diagnosis of sarcoidosis was suspected by the association of clinical, biochemical and radiologic features of sarcoidosis, and confirmed by histology showing non-caseating granulomas. Diagnosis of liver involvement was based on the presence of non caseating granuloma on liver biopsy. Others causes of granulomatous hepatitis (mainly tuberculosis, primary biliary cirrhosis, viral hepatitis B and C and drugs) were investigated and excluded. Clinical, biochemical, imaging and pathological features were detailed. Treatment was specified as well as the outcome. Response to treatment was evaluated after 12 months of therapy. Complete response was defined by normalization of biochemical markers, partial response by biochemical improvement without normalization, and refractoriness with stagnation or worsening biochemical parameters.

## Results

### Clinical features

We included 7 cases of systemic sarcoidosis with hepatic involvement represented by 5 women and 2 men. The average age was 43 years (extremes 31-59 years). The hepatic localization revealed sarcoidosis in 5 cases: 4 patients presented with right upper-quadrant abdominal pain; in 1 case, sarcoidosis was revealed by abnormal liver tests discovered fortuitously trough a regular blood test. In the two latter cases, liver involvement complicated a sarcoidosis already known. Hepatomegaly was observed in all patients, and splenomegaly was present in 2 patients, concomitant with BCS in one case. None of our patients had jaundice, pruritus, liver failure, ascites or hepatic encephalopathy.

### Biochemical data

All patients had abnormal serum liver function test reflected by anicteric cholestasis. The mean alkaline phosphatase level (APL) level was 234 UI/l (Normal range (NR) 40-129 U/L), that of ɤGlutamyl Trans peptidase (ɤGT) was 119U/L (NR 2-30 U/L). It was associated with elevated aminotransferases less than 2 times the upper limit of normal in 2 cases. International normalized ratio (INR), albumin level and platelet count were within normal range in all cases. Serum angiotensin converting enzyme (ACE) was elevated in 5 patients with an average of 187 UECA (extremes: 6-283 UECA; NR 12-68). None of our patients had increased calcium levels. An elevated acute phase reactants was noted in all cases.

### Imaging findings

Homogeneous liver enlargement was found on ultrasound (US) or computerized tomography (CT) scan in all cases. Abdominal lymph node enlargement were detected in 2 cases, 3 patients had splenomegaly with presence of low attenuation nodules in one patient. Hepatic vein were not demonstrated in 2 patients in combination with hypertrophied caudate lobe and collateral circulation, which was suggestive of primary BCS.

### Pathological findings

Liver biopsy, performed in all patients, disclosed sarcoidosis lesions with the presence at the portal areas of small non caseating granulomas in all cases. The liver architecture was preserved in four patients. Extensive fibrosis was observed in two patients. Bile duct injury was observed in two patients, consisting in lymphocytic cholangitis and cholangiolar hyperplasia.

### Extra-hepatic manifestations

Systemic signs of fatigue and weight loss were found in four patients. Pulmonary involvement was present in all patients and was symptomatic in the form of a dry cough or dyspnea. One patient had monoarthitis with inflammatory but sterile synovial fluid. Involvement of gastrointestinal tract was found in 2 patients and was located in stomach. These patients complained of atypical abdominal pain. Upper endoscopy disclosed congestive ulcerated gastropathy in one case and atrophic fundic gastropathy in the other case, biopsies showed non caseating granuloma. Two cases of skin sarcoidosis were observed, consisting of sarcoid nodule and nonspecific erythematous lesion of scalp and forehead confirmed by histology for the latter. Ocular involvement was found in 2 patients: sicca syndrome and anterior uveitis. None of our patient had neurological involvement. Two patients were screened for cardiac sarcoidosis and no case of cardiac involvement had been highlighted. Analysis of a biopsy sample of minor salivary gland had shown non caseous granulomas in 4 patients. Screening for an underlying prothrombotic condition was negative in the 2 patients with BCS, which was finally considered as a complication of hepatic sarcoidosis. Clinical and laboratory characteristics of patients are summarized in Table 1, Table 2.

**Table 1:** Patients clinical characteristics

	1	2	3	4	5	6	7
Age (years)	45	58	36	59	36	42	32
Sex	F	F	H	F	G	F	F
Presentation Signs	Abd Pain	Abd Pain	Abd Pain Dyspnea	Abd Pain	Abd Pain	Asymptomatic Cholestasis	Abd Pain
Pulmonary Manifestations Clinical	Dyspnea	-	Dyspnea	Cough	-	Dyspnea Cough	-
Radiological Stage	1		0	0	1	1	1
Pulmonary function	Normal	1	-	Normal	-	-	-
Broncho-alveolar lavage	-	Restrictive Syndrome	>5	>5	Macrophagic	-	-
CD4/CD8		>5			Formula		-
Nodes Peripherical	-	-	+	-	-	-	-
Abdominal	-	-	+	+	-	+	-
Cutaneous Manifestations	-	Sarcoid nodules	-	-	-	Scalp erythematous lesion	-
Ocular manifestations	-	uveitis	-	-	Sicca Syndrome	-	-
Hepatomegaly	+	+	+	+	+	+	+
Splenomegaly	-	+	-	-	+	+	-
							

F: female, M: male, Abd: abdominal

**Table 2:** Biochemical parameters and course with treatment

	1	2	3	4	5	6
Liver Tests	PAL 2.7N GGT 7N ALT 1.5N	PAL 2N GGT 3N ALT N	PAL N GGT 2N ALT N	PAL N GGT N ALT N	PAL N GGT 3N ALT N	PAL 2.3N GGT 4N ALT N
Abdominal Ultrasound	HMG	HMG	HMG	HMG	HMG Hypertrophy seg 1 Non viewed hepatic vein SMG	HMG Hypertrophy seg 1 Non viewed hepatic vein SMG
Abdominal CT	-	HMG SMG	HMG Lymph nodes enlargement	HMG Lymph nodes enlargement	Idem	Idem
Liver Histology	Granuloma No fibrosis	Granuloma Portal Fibrosis	Granuloma No Fibrosis	-	Granuloma Extensive fibrosis	Granuloma Extensive fibrosis Cholangiolar hyperplasia
Treatment	1-CS 0,5mg/kg 2-AntimalariaL/UDCA	CS 0,5mg/kg	CS 0,5mg/kg	CS 0,5mg/kg	UDCA 15 mg/kg	CS 1mg/kg
Evolution	1- No Response 2-Total Response	Partial Response	Total Response	Recent Introduction	Lost Sight	Recent Introduction

N: upper limit of normal, HMG: Hepatomegaly, SMG: Splenomegaly, Seg: Segment, CS: Corticosteroids, CT: computed tomography

### Treatment

All patients were treated. Five were treated by corticosteroid (prednisone 0,5-1mg/kg daily) and 2 with ursodeoxycholic acid (UDCA) (13-15mg/kg daily). Evolution after 12 months of corticosteroid therapy was characterized by complete response in one case, partial response in another case and corticosteroid refractoriness in one case. In two cases, corticosteroid therapy was introduced for less than 1 month, not allowing assessment of response. Antimalarials in combination with UDCA were used successfully in a patient with steroid-resistant liver disease. For the 2 patients treated with UDCA: on had not improved liver tests and the second were lost sight. Anticoagulation by was given in the two patients with BCS, with INR target between 2 and 3.

## Discussion

We described a case series of liver sarcoidosis. Although liver involvement is common in sarcoidosis, it rarely causes symptoms and may remain undiagnosed in many cases. For these reasons treatment is not codified and many patients are treated pragmatically with corticosteroids or receive no treatment [2]. Nevertheless, in a minority of cases, hepatic sarcoidosis cause severe complications such as severe cholestatic jaundice, portal hypertension, BCS, cirrhosis and lead to end stage liver disease. Consequently, it is important to give an accurate description of clinical presentation, biochemical and imaging findings of hepatic sarcoidosis in order to identify which patients will need close monitoring and treatment. In the present case series, liver involvement revealed sarcoidosis in most cases. This situation is uncommon, and usually, hepatic sarcoidosis is most commonly diagnosed in the staging of a known sarcoidosis. Indeed, in previous series, sarcoidosis was already known between 33% and 80% of patients [3–6]. Our findings may be related to the fact that all patients were recruited from a department of Hepatology, which is a source of bias. This may also explain why almost all patients were symptomatic for liver involvement whereas in the literature symptoms are present in approximately 12% of cases [7]. According to a recent review, during the initial evaluation of sarcoidoisis, systematic liver function tests, abdominal US and CT are recommended in order to highlight liver involvement [8]. Biopsy is indicated in case of moderate or severe abnormalities on liver tests [8]. Abnormality in liver tests are encountered in 20-40%, they usually reveal a cholestatic pattern [9]. Increased ALP, ɤGT, bilirubin (usually <5mg/L) and slightly elevated aminotransferase levels are typical laboratory findings [9]. This pattern is in agreement with our findings, were cholestasis was observed in all patients with associated elevated aminotransferase levels in two cases. Bilirubin level was slightly increased in one case only. Contributing factors for cholestasis seems to be the progressive interlobular bile duct injury due to inflammatory infiltration of basement membranes and portal granuloma formation. It has been proposed that the latter results in fibrosis of portal tracts, vanishing bile ducts and ductopenia [9]. This histological picture resembles primary biliary cirrhosis, but positivity of antimitochondrial antibodies, absence of extra-hepatic granuloma, less numerous granulomas in the live histology, directs the diagnosis toward primary biliary cirrhosis.

Usually, liver sarcoidosis rarely leads to severe liver damage, but in the present series, 2 patients had extensive fibrosis and BCS. There are few case-reports of BCS complicating hepatic sarcoidosis. Thrombosis of hepatic vein may be related to external compression by granuloma or granulomatous involvement of the hepatic vein resulting in narrowing of the lumen and ensuing stasis [10]. Evidence based guidelines for the treatment of hepatic sarcoidosis are lacking. Patients with severe extra-hepatic organ involvement generally require treatment and don't raise difficulties. Controversy arises when hepatic sarcoidosis is the main presenting feature, while extra-hepatic involvement is not life threatening and doesn't require treatment. This situation corresponds to the majority of our patients. Generally, the clinical course for sarcoidosis varies. Independently of the localization, in half of cases, disease resolves spontaneously within 2 years [8]. Likewise, isolated histological lesions do not imply the startup of a corticosteroid treatment, hepatic and splenic locations discovered incidentally on imaging may have an evolution spontaneously favorable [11]. On the other hand, debate exists about whether treatment can change the outcome of sarcoidosis, particularly fibrosis [8]. Regarding liver sarcoidosis, although corticosteroids may improve clinical and laboratory parameters, they do not reverse histological changes and preclude progression and development of portal hypertension. In our series, efficacy of corticosteroid was insufficient. Moreover, among indications of systemic corticosteroid treatment, some authors specify “severe liver involvement” [12]. We suppose that severe liver disease correspond to symptomatic patients with jaundice, pruritus, those with severe cholestasis or liver impairment. The increased awareness of long term side-effects of corticosteroids, and the emergence of new drugs, has changed the treatment of sarcoidosis [8]. Alternative pharmacological options have been assessed. In a retrospective cohort of 17 patients, UDCA at a dose of 13-15mg/kg daily had a positive effect on pruritus and liver biochemistry, supporting the empirical use of UDCA in the treatment of liver sarcoidosis [2]. In our series, antimalarials in combination with ursodeoxycholic acid were used successfully in one a patient with steroid-resistant liver disease. The importance of TNF alpha in granuloma formation led to proposing this cytokine as a therapeutic target in sarcoidosis. Infliximab has been proposed in the case of sarcoidosis with steroid refractory liver localization. It seems beneficial to the clinical manifestations but not to the biochemical disturbances liver [13]. Immunosuppressants are sometimes an alternative in the case of high-dose corticosteroid but their liver toxicity remains problematic. In case of liver failure, some patient may require liver transplantation [14].

## Conclusion

In this study, liver involvement revealed systemic sarcoidosis and was symptomatic in all cases. Serum liver function disclosed cholestasis in all cases and 2 patients had severe fibrosis at liver biopsy. Usually, hepatic involvement in sarcoidosis is clinically latent. However, it must be screened for in all patients with systemic sarcoidosis using liver function tests and imaging given the risk of severe liver damage. Liver biopsy, not without complications should only be performed in the presence of the disturbance liver biology. The severity of liver disease depends essentially on the occurrence of complications such as portal hypertension and cirrhosis. Treatment is not systematic, and still based on corticosteroid therapy but does not always allow remission. Its indication depends on the importance of liver damage and other disease localizations. In the absence of prospective randomized controlled trials, the efficacy of UDCA need to be proven.

### What is known about this topic

- Clinical, biochemical and morphologic manifestations.

### What this study adds

- BCS as a rare presentation of hepatic sarcoidosis;
- Treatment results and perspectives.
